# The role of cellular adhesion molecules in virus attachment and entry

**DOI:** 10.1098/rstb.2014.0035

**Published:** 2015-02-05

**Authors:** David Bhella

**Affiliations:** Medical Research Council—University of Glasgow Centre for Virus Research, Sir Michael Stoker Building, Garscube Campus, 464 Bearsden Road, Glasgow G61 1QH, UK

**Keywords:** virus, receptor, cell entry, cryo-electron microscopy, cell adhesion

## Abstract

As obligate intracellular parasites, viruses must traverse the host-cell plasma membrane to initiate infection. This presents a formidable barrier, which they have evolved diverse strategies to overcome. Common to all entry pathways, however, is a mechanism of specific attachment to cell-surface macromolecules or ‘receptors’. Receptor usage frequently defines viral tropism, and consequently, the evolutionary changes in receptor specificity can lead to emergence of new strains exhibiting altered pathogenicity or host range. Several classes of molecules are exploited as receptors by diverse groups of viruses, including, for example, sialic acid moieties and integrins. In particular, many cell-adhesion molecules that belong to the immunoglobulin-like superfamily of proteins (IgSF CAMs) have been identified as viral receptors. Structural analysis of the interactions between viruses and IgSF CAM receptors has not shown binding to specific features, implying that the Ig-like fold may not be key. Both proteinaceous and enveloped viruses exploit these proteins, however, suggesting convergent evolution of this trait. Their use is surprising given the usually occluded position of CAMs on the cell surface, such as at tight junctions. Nonetheless, the reason for their widespread involvement in virus entry most probably originates in their functional rather than structural characteristics.

## 1. Introduction

Fundamentally, viruses are infectious nucleic acids that have evolved efficient mechanisms for shuttling their genomes between the host cells that they depend upon for replication. A key stage in the viral replication cycle is cell entry. To initiate infection, all viruses must traverse the host-cell's plasma membrane and in many cases a cell wall. For those viruses that infect animals, the first stage of this process is attachment to a cell-surface macromolecule, the viral receptor. There is considerable interest in understanding the virus–receptor interaction at the structural level. As the first step in the infection process, viral attachment represents an attractive target for intervention. The process of receptor engagement leads to initiation of the internalization pathway. Furthermore, receptor binding is frequently the trigger for conformational changes in the virion itself. These structural rearrangements are thought to initiate the uncoating process—the controlled, targeted release of the genome to the site of replication.

## 2. Viral entry pathways

Viruses employ diverse entry pathways following attachment. The host-cell plasma membrane presents a significant barrier, penetration of which may be facilitated by the presence of a viral membrane or envelope. Enveloped viruses acquire their membrane from the host either by budding from the plasma membrane of an infected cell, or by budding into cellular compartments. In either case, the membrane will bear viral glycoproteins that mediate attachment and entry. These functions may be performed by a single glycoprotein or may be divided between two or more. Following attachment, the glycoprotein responsible for mediating cell entry is activated to become fusogenic, undergoing conformational changes resulting in insertion of a hydrophobic ‘fusion-peptide’ into the host-cell plasma membrane. Further structural rearrangements then bring the viral and cellular membranes together leading to the formation of a fusion pore. The contents of the virion, including the encapsidated viral genome (nucleocapsid) are then delivered into the cytosol.

Not all enveloped viruses initiate fusion at the plasma membrane. Influenza viruses, for example, enter through the clathrin-mediated endocytic pathway [1]. Acidification of the late endosome triggers the activity of the viral fusion protein haemagglutinin, leading to release of the virion contents into the cytosol [2]. Endocytosis is also the most common entry mechanism for non-enveloped (proteinaceous) viruses; however, the manner in which these viruses leave the endosome is, in general, poorly understood. It is thought that this is accomplished either by the formation of a pore in the endosomal membrane through which the genome is ejected, or destruction of the endosomal membrane by viral-encoded gene products [3,4].

## 3. Receptor usage and viral tropism

Receptor usage is a key factor in defining tropism in many viruses; for example, influenza viruses bind to sialic acid moieties on the apical surfaces of epithelial cells in the respiratory tract of mammals or the gut of avian species. These are the primary sites of viral replication in the respective hosts. Those viruses that infect humans have evolved to bind α2,6 sialic acid which is found primarily in the upper respiratory tract. The haemagglutinin protein of avian viruses, on the other hand, binds to α2,3 sialic acid, which is the predominant form in the avian gut epithelium [5]. Thus, evolving to bind differently linked sialic acids is thought to be one important step required for avian viruses to transmit readily between human hosts. Evolution of receptor usage is therefore a key event that may lead to emergence of new pathogens with altered pathogenicity or host ranges.

## 4. Viral entry via multiple receptor molecules

Binding to sialic acid is a widely used strategy for attachment to the cell surface in diverse groups of viruses. Indeed, several classes of receptor molecule have been identified that are repeatedly found to be used by apparently unrelated viral species. These include integrins and cell-adhesion molecules that are members of the immunoglobulin-like superfamily (IgSF CAMs), the latter being the main focus of this review. Interestingly, two quite different viruses, feline calicivirus (FCV) and reovirus, have been found to employ both sialic acid and the IgSF CAM junctional adhesion molecule A (JAM-A) [6–9], while the picornavirus encephalomyocarditis virus binds sialic acid and a different IgSF CAM, vascular cell-adhesion molecule 1 (VCAM-1) [10,11]. A requirement for more than one receptor molecule is not uncommon and many viruses have evolved multi-step attachment processes. One extreme example is hepatitis C virus (HCV), which has been shown to require several molecules for cell entry including heparan sulfate [12], liver specific intercellular adhesion molecule-3-grabbing non-integrin (L-SIGN) or dendritic cell intercellular adhesion molecule-3-grabbing non-integrin (DC-SIGN) [13–15], low-density lipoprotein receptor (LDL-R) [16,17], transferrin receptor 1 (TfR1) [18], Niemann-Pick C1-like protein 1 (NPC1L1) [19], scavenger receptor class B type I (SR-B1) [20], the tetraspanin CD81 [21] and the tight-junction components claudin-1 (CLDN-1) [22] and occludin (OCLN) [23]. SR-B1, CD81, CLDN-1 and OCLN are considered the minimal requirements for cell entry, while attachment to L-SIGN is postulated to confer tissue tropism *in vivo*. Lying at the centre of the HCV entry pathway is the interaction between the viral envelope glycoprotein E2 and CD81, which triggers actin-dependent trafficking of the virus to tight junctions where it comes into contact with CLDN-1 and OCLN, leading to viral entry by endocytosis.

Trafficking of virus to tight junctions from the apical cell surface was first demonstrated for group B coxsackie viruses (CVBs). Many CVBs bind to coxsackievirus and adenovirus receptor (CAR—an IgSF CAM) as well as the complement control protein decay-accelerating factor (DAF, also known as CD55). Clustering of DAF molecules by virus attachment to the apical cell surface stimulates remodelling of the actin cytoskeleton by Abl kinase. This, in turn, leads to delivery of the virus to the tight junction where entry occurs by caveolin-mediated endocytosis [24].

## 5. The structure and functions of Ig-like cell-adhesion molecules

**Figure 1.**
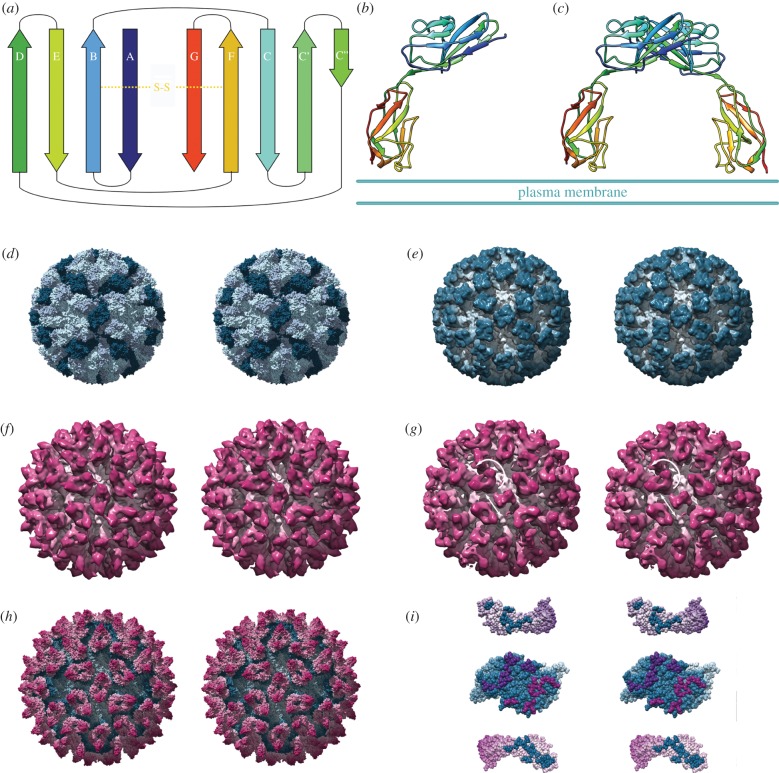
The immunoglobulin superfamily of proteins are characterized as having domains of between seven and nine beta-strands arranged in two antiparallel sheets that form a sandwich structure, stabilized by a conserved disulfide bridge (*a*; rainbow coloured from the N-terminus (blue) to the C-terminus (red)). The crystal structure of a typical IgSF CAM—human JAM-A (*b*; PDB 1NBQ [34]) reveals that the two-domain molecule exists as a dimer in solution that is thought to represent the native structure at tight junctions (*c*). The ribbon diagrams are rainbow coloured across two domains, hence the colours of individual strands in (*b*) and (*c*) do not correspond to those in (*a*). Caliciviruses such as feline calicivirus (FCV) are RNA containing viruses that have a *T* = 3 icosahedral capsid (*d*; PDB 3M8L) [35]. This is composed of 180 capsid proteins (VP1) arranged as two classes of dimer: AB dimers (light and mid-blue) and CC dimers (dark blue). Cryo-electron microscopy of FCV (*e*) decorated with a soluble fragment of feline JAM-A (*f*) reveals that the receptor binds to the tip of the protruding domain of VP1. Receptor engagement induces conformational changes in the viral capsid such that the AB dimer rotates 15° anticlockwise and the CC dimer tilts away from the twofold symmetry axis (*g*; arrows). Docking high-resolution coordinates to the three-dimensional reconstruction led to the calculation of a quasi-atomic resolution map of the virus—receptor complex (*h*; FCV coloured blue, fJAM-A coloured magenta) that allowed the identification of putative contact residues (*i*). The VP1 AB dimer is viewed from the virus exterior, fJAM molecules viewed as if peeled away from the capsid surface and rotated 180^o^ [36]. Panels (*d*–*i*) are presented as wall-eyed stereo pairs. (*e*–*i*) adapted from [36].

An intriguing aspect of viral receptor usage is the widespread exploitation of cell-surface glycoproteins that are found predominantly in intercellular junctions of polarized cells. Perhaps the most widely used class of adhesion molecule is the IgSF CAMs (table 1). The immunoglobulin-like superfamily of proteins is characterized as consisting of seven to nine anti-parallel beta-strands that form two beta-sheets in a Greek-key motif, having a barrel shape. The superfamily is subdivided according to the number of beta-strands and topological similarities to the constant (c) or variable (v) chains of antibodies (V, C1, C2, I). Figure 1*a* shows the topology of the V-set Ig-like fold (reviewed in [37]).

**Table 1.** Diverse groups of viruses have been shown to bind to immunoglobulin-like superfamily cell-adhesion molecules to gain entry to the host cell. Both DNA and RNA viruses exploit this class of molecules, as do enveloped and proteinaceous viruses.

virus	receptor name	abbreviation	references
*proteinaceous viruses*			
*Adenoviridae*			
human adenovirus C	coxsackievirus–adenovirus receptor	CAR	[25]
*Caliciviridae*			
feline calicivirus	junctional adhesion molecule A	JAM-A	[8]
*Picornaviridae*			
coxsackie A virus type 21	intercellular adhesion molecule 1	ICAM-1/CD54	[26]
coxsackie B virus	coxsackievirus–adenovirus receptor	CAR	[25]
encephalomyocarditis virus	vascular cell-adhesion molecule 1	VCAM-1/CD106	[10]
major receptor group rhinovirus	intercellular adhesion molecule 1	ICAM-1/CD54	[27]
poliovirus	poliovirus receptor	PVR/CD155	[28]
*Reoviridae*			
reovirus	junctional adhesion molecule A	JAM-A	[6]
*enveloped viruses*			
*Coronaviridae*			
mouse hepatitis virus	carcinoembryonic antigen-related cell-adhesion molecule 1	CEACAM-1/CD66a	[29]
*Herpesviridae*			
herpes simplex virus	nectin-1 nectin-2	HvecC/CD111 HvecB/CD112	[30,31]
*Paramyxoviridae*			
measles virus	nectin-4		[32]
*Rhabdoviridae*			
rabiesvirus	neuronal cell-adhesion molecule 1	NCAM-1/CD56	[33]

JAM-A is a prototypic tight junction associated IgSF CAM expressed on epithelial and endothelial cells as well as on leucocytes and platelets [38]. It comprises two extracellular Ig-like domains, a single transmembrane region and a short cytoplasmic tail. X-ray crystallography of an ectodomain soluble fragment of human JAM-A reveals that the N-terminal, membrane-distal D1 domain has nine beta-strands and is therefore classified as similar to the antibody variable domain (V-set). The membrane proximal D2 domain, on the other hand, is classed as I-set, having only eight strands (figure 1*b*).

Analytical ultracentrifugation analysis of recombinant soluble murine JAM-A revealed that it forms homodimers. Both murine and human JAM-A crystal structures show a similar non-covalent interaction between the membrane-distal D1 domains at the GFCC’ face, and this is thought to represent the dimeric state of JAM-A at the cell surface (figure 1*c*). Homotypic and heterotypic interactions with JAM-A and other adhesion molecules, respectively, on adjacent cells, are then thought to regulate tight-junction formation and facilitate leucocyte transmigration [34,39].

## 6. Feline calicivirus binding to JAM-A

FCV is one of only a handful of tractable models for the *Caliciviridae*, a family of positive-sense RNA containing icosahedral viruses, which includes norovirus, the cause of winter vomiting disease. These small proteinaceous viruses assemble a *T* = 3 icosahedral capsid from 90 dimers of a single major capsid protein VP1 (figure 1*d*) [35]. The virion is characterized by the presence of protruding (P) domains that give rise to the appearance of cups on the surface of the particles when viewed by negative stain electron microscopy; hence their name, which derives from the Latin *calyx*.

FCV has been shown to bind to both N-linked α2,6 sialic acid and feline JAM-A (fJAM-A) [8,9]. Both molecules are important for cell entry, which is by clathrin-mediated endocytosis and requires acidification of the endosome [40].

fJAM-A is the only protein receptor to be identified for any member of the *Caliciviridae*. Experiments demonstrated that transfection of the fJAM-A gene into non-permissive cells rendered them susceptible to FCV infection, while antibodies raised against fJAM-A blocked infection [8]. To investigate the structural basis for fJAM-A receptor engagement, we used cryogenic electron microscopy (cryoEM) combined with computational three-dimensional image reconstruction to determine the structure of purified FCV particles decorated with a soluble fragment of fJAM-A (figure 1*e*–*g*) [36,41]. This revealed that the fragment binds to the outer face of the capsid P domain. Two fJAM-A molecules lie in a head-to-tail arrangement about the twofold symmetry axis of each VP1 dimer. Docking high-resolution coordinates for FCV and fJAM-A—derived from crystallographic analysis and homology modelling respectively—led to the synthesis of a quasi-atomic resolution model of the receptor decorated virion (figure 1*h*). This showed that the membrane-distal D1 domain was primarily responsible for the interaction, and key residues in both virus and receptor that are involved in viral attachment were identified (figure 1*i*). Interestingly, the soluble fJAM-A fragment did not bind to the capsid in the dimeric state seen in the structures of human and murine forms solved by X-ray crystallography. The oligomeric state of fJAM-A used in our study is not known; however, the site of virus attachment to JAM-A does not suggest that viral binding would directly compete with the homodimer interaction. The FCV-binding site on JAM-A resides in the beta-sheet comprising strands ABE while the homodimer interface is at the opposite GFCC’ face. It may, however, be the case that binding to FCV disrupts the JAM-A homodimer by inducing a structural change in the receptor.

In our study, substantial conformational changes in the viral capsid protein were seen upon receptor binding. The P-domain of the AB dimer was seen to rotate 15° counterclockwise, while at the CC dimer the P-domain tilted away from the icosahedral twofold symmetry axis. We hypothesize that these changes in virion conformation may reflect the early stages of uncoating, priming the capsid for subsequent genome release.

## 7. Picornavirus attachment

The *Picornaviridae*, like the *Caliciviridae*, are small icosahedral, non-enveloped, positive-sense RNA-containing viruses. Many viruses in this family have been found to have IgSF CAM receptors. Interestingly, these receptors also induce profound conformational changes in the virion upon binding, destabilizing the capsid and leading to genome release. However, those viruses that bind to non-IgSF receptors do not appear to undergo such receptor-induced rearrangements.

**Figure 2.**
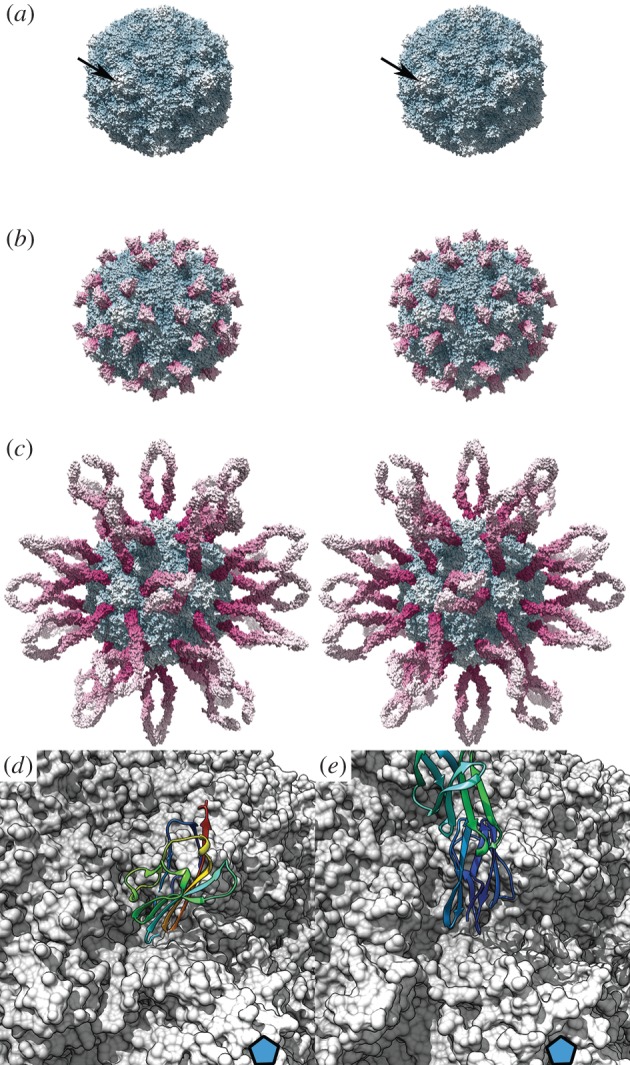
Picornaviruses are small proteinaceous icosahedral viruses, many of which have pronounced star-shaped mesas at their fivefold symmetry axes (*a*; arrow) that are surrounded by canyons which contains the receptor-binding sites for IgSF CAMs. Coxsackie B virus type 3 binds to the coxsackievirus and adenovirus receptor (*b*; CAR domain 1 only is shown, figure generated using PDB 1COV, 1KAC based on 1JEW [42–44]). Coxsackie A virus type 21 binds to intercellular adhesion molecule 1 (ICAM-1) (*c*; PDB 1Z7Z [45]). Both molecules are oriented perpendicular to the capsid surface. A close up view of the interaction (receptors shown as ribbon diagrams) shows that CAR (*d*) and ICAM-1 (*e*) bind to the canyon in a similar but not identical orientation (fivefold symmetry axis indicated by a blue pentagon). (*a*–*c*) Stereoscopic views in which virus is radially coloured blue-white and receptor is radially coloured magenta-white. Panels (*d*) and (*e*) are monoscopic and the virus surface is shown in grey; the receptor is shown as a rainbow-coloured ribbon diagram.

Picornaviruses have generally well-conserved virion morphology. The icosahedral capsid assembles from four structural proteins designated VP1–4. VP1–3 occupy positions conventionally taken by multiple copies of a single protein species in a *T* = 3 icosahedral lattice; thus picornaviruses are described as *pseudo T* = 3 or P3. Most picornaviruses have pronounced star-shaped mesas at their icosahedral fivefold symmetry axes that are surrounded by deep canyons (figure 2) [46]. CryoEM studies of picornavirus–CAM complexes show that the tip of the membrane-distal Ig-like domain inserts into the canyon such that the receptor is oriented more or less perpendicular to the capsid surface [42,45]. This manner of receptor engagement is rather different to that seen in FCV, which binds to one side of the D1 domain. Surprisingly, however, detailed comparison of the interactions between picornaviruses and their respective IgSF CAM receptors revealed that they bind to different faces of the D1 domain [47].

## 8. Evolution of virus–receptor interactions

**Figure 3.**
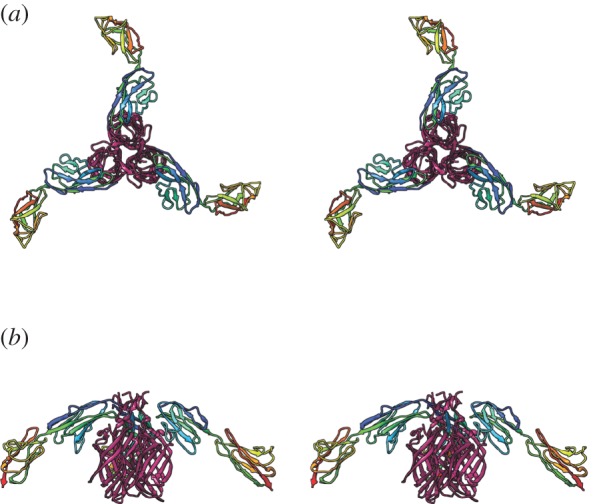
(*a*,*b*) Views of the interaction between JAM-A (rainbow coloured) and the reovirus attachment protein σ-1 (magenta; wall-eyed stereo pairs—PDB 3EOY, 1NBQ [34,49]).

Structural analysis of viral attachment proteins complexed to Ig-like SF CAMs provides us with detailed descriptions of the first step of the infectious process. In all cases studied so far, the virus binds to the V-set membrane-distal D1 domain. It has been suggested that viruses engage Ig-like SF molecules in a manner that parallels immunoglobulin pathogen recognition [48]. The above-mentioned comparison of interactions in the *Picornaviridae* and our analysis of FCV JAM-A binding are at odds with this, however, suggesting that viruses do not exploit a common binding site. The FCV-binding site of fJAM-A comprises residues in strands A, B and E, while entry of reoviruses is mediated by an attachment protein σ-1, which engages the dimer interface of monomeric JAM-A at residues in the C and C’ beta-strands (figure 3) [49]. Thus, opposite faces of the D1 domain are required for entry of these two viruses. In the case of reovirus attachment it is possible that virus binding may directly disrupt JAM-A dimers.

Reo- and caliciviruses are quite distinct classes of viruses. The *Orthoreovirinae* are large proteinaceous double-stranded RNA containing viruses with a complex multi-layered capsid. Protruding from each fivefold vertex are trimeric fibres of the attachment protein σ-1 that terminate in a globular knob domain comprising three beta-barrels. Evolution of JAM-A and sialic acid binding in reo- and caliciviruses is therefore most probably a product of convergent evolution. The fact that IgSF CAM binding is widely observed in both proteinaceous and enveloped viruses also argues that the exploitation of such molecules is a highly advantageous capability that has emerged repeatedly and independently.

**Figure 4.**
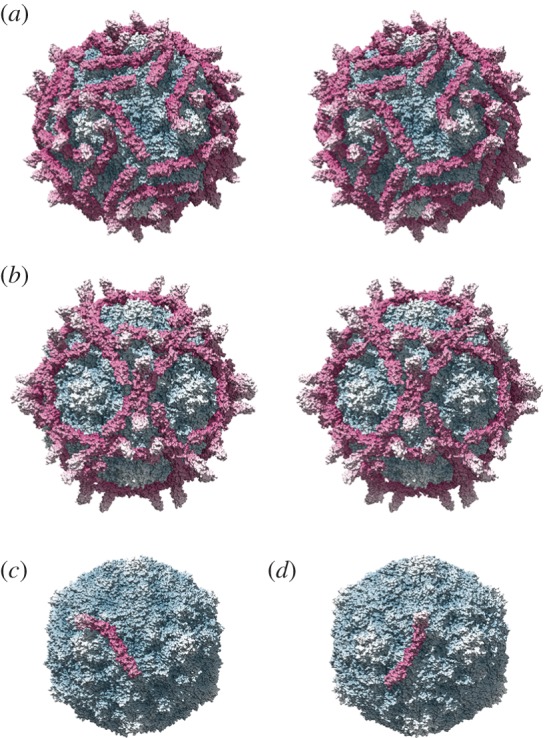
Evolution of virus–receptor interactions. Coxsackie B virus type 3 (CVB3) binds to the complement control protein decay-accelerating factor (DAF or CD55) at the apical cell surface prior to trafficking to tight junctions where entry is mediated by the coxsackievirus and adenovirus receptor (CAR). Comparison of the CVB3–DAF interaction (*a*; PDB 1COV, 3J24 [50]) with that of the distantly related picornavirus echovirus type 12 (EV12) (*b*; PDB 2C8I [51]) shows a markedly different receptor orientation. The structure is more easily interpreted when only a single DAF molecule is shown. CVB3 (*c*) binds primarily to domain 2 of DAF. EV12 binds predominantly domain 3 (*d*); moreover, there is approximately 90° rotation in the orientation of the two molecules on the capsid surface. These two viruses have quite different receptor interactions but have probably evolved from a common DAF-binding picornavirus ancestor. In each panel, the virus is radially coloured blue-white; DAF-CD55 is radially coloured magenta-white.

Virus–receptor interactions in animal viruses are subject to continual selective pressure in the face of immune surveillance. A consequence of this is that the outer surfaces of virus particles are characterized by the presence of elaborate loop structures and hypervariable regions that disrupt antibody-mediated neutralization and serve to camouflage the more highly conserved receptor-binding site. Under these circumstances it is likely that the binding site itself will nonetheless undergo constant modification. It iseasy to imagine how, through mutation of the capsid or attachment proteins, the receptor may ‘walk’ over the surface of the virus. Likewise, the virus may, over time, evolve to bind a different face of the receptor molecule or even to bind a different structurally related molecule. Strong evidence for this can be seen in the diverse interactions displayed by distantly related picornaviruses that bind to the complement control protein DAF. DAF (also known as CD55) is not a member of the immunoglobulin-like superfamily of proteins but displays a striking divergence of binding modes by viruses that exploit it as a receptor. In research published by ourselves and others, cryoEM analysis of echovirus type 12 (EV12) and coxsackie virus type B3 (CVB3) decorated with DAF revealed a marked difference in the orientation of the receptor molecule on the virion surface (figure 4) [50,51]. DAF comprises four short-consensus repeat domains. CVB3 binding occurs primarily at SCR2 while EV12 binds to SCR3. Moreover, there is a rotation of almost 90^o^ in the position of the DAF molecule relative to the capsid surface. It has been suggested that the differences in DAF binding in these two viruses is the product of convergent evolution to bind a common receptor. Given the high mutation rates in RNA containing viruses and the above-mentioned selective pressure, however, divergent evolution from a common DAF binding ancestor would seem equally or more plausible.

As we have seen, IgSF CAM binding occurs widely among diverse classes of viruses, suggesting convergent evolution. On the other hand, we can see how a process of continual evolution in the face of immune surveillance may have led to diversification of canyon-binding IgSF CAM interactions within the *Picornaviridae.* Both convergent and divergent evolution of this trait among different viruses argues that there is a strong selective pressure to gain and retain a capability to bind these molecules. As noted above, however, evidence for a conserved binding motif is not compelling. Thus, the requirement to bind to cell-adhesion molecules may originate in their functional properties despite the apparent inconvenience of requiring the virus to traffic across the cell surface to intercellular junctions to effect cell entry. Recent research in this area is beginning to provide insights into the possible reasons for the persistence of this phenomenon.

## 9. Disruption of CAR homotypic interactions leads to endocytosis

Group C adenoviruses bind to CAR in a manner that closely parallels the reovirus JAM-A interaction. A trimeric fibre knob protein engages the normally dimeric CAR molecule at an interface that overlaps its dimerization site. The recent finding by Salinas *et al.* [52] that disruption of the CAR homodimer by adenovirus fibre knob protein stimulates endocytosis provides one possible explanation for the conservation of IgSF CAM binding among diverse groups of viruses. Endocytosis is an important aspect of IgSF CAM function, serving as a means of intracellular signal transduction. Moreover, depletion of CAMs from the cell surface is critical in regulation of cell migration. Thus exploitation of this important functional quality of IgSF CAMs could allow viruses to gain entry to the cell interior. Several viruses that engage IgSF CAMs are, however, enveloped and postulated to enter by fusion at the plasma membrane. Thus triggering endocytosis may not be the sole reason for IgSF CAM usage, and further work is necessary to establish whether those enveloped viruses undergo endocytosis at intercellular junctions.

## 10. JAM-A facilitates dissemination of reovirus infection

Much of our understanding of virus entry derives from studies of viruses grown in cell culture. Our comprehension of the complexities of virus behaviour within tissue or the whole organism is therefore limited. To investigate the role of JAM-A in reovirus infectious processes, Antar *et al.* [53] investigated pathogenesis in JAM-A null mice. Animals were perorally inoculated with a neurotropic strain of the virus. The primary site of infection, the intestine, was infected normally; however, JAM-A^−/−^ mice showed no sign of neurological disease. In these studies, virus replication at sites of secondary infection was significantly reduced as a consequence of a failure of the virus to enter the blood-stream. Neural spread was, however, unaffected. The authors suggest that JAM-A plays a crucial role in establishment of viraemia, either by facilitating infection of endothelial cells leading to release of virus from apical cell surfaces into the bloodstream or receptor-specific transcytosis of virus across endothelial cells. An alternative hypothesis is that dissemination of virus through the bloodstream might be mediated by infection of blood leucocytes.

## 11. Viral exploitation of IgSF proteins expressed on cells of the immune system

In addition to the above-mentioned exploitation of IgSF CAMs as viral receptors, there are several viruses that have evolved specifically to infect cells of the immune system through engagement of other IgSF molecules. These include T-cell membrane protein 1 (TIM-1) which is bound by Ebola, dengue and hepatitis A viruses [54–56], the HIV receptor CD4 [57], and signalling lymphocyte-activation molecule (SLAM) [58], which is exploited by measles virus. The ability to infect migratory cells of the immune system is a strategy that allows viral dissemination within the host without danger of exposure to the adaptive immune response. It seems plausible, then, that the viral strategy of infecting via IgSF CAM mediated entry may also confer this advantage. Immune cells display IgSF CAMs to mediate transmigration through tissues. In the case of FCV and reovirus infection of the respiratory and intestinal epithelium, respectively, leucocytes responding to the viral attack may themselves become infected and facilitate dissemination to secondary sites of infection.

## 12. Summary

Many diverse groups of viruses bind to IgSF CAMs at the cell surface to mediate cell entry. Structural analyses of virus receptor complexes do not, however, indicate a common mode of binding, suggesting that this phenomenon is not a simple exploitation of the adhesive properties of these molecules. Cell-adhesion molecules play a critical role in maintaining tissue integrity and also mediating migration of immune cells. This is achieved through control of surface expression levels. Endocytosis of CAMs is the primary means of modulating surface expression, and there is evidence that this is mediated by disruption of the CAM homodimer interface. Exploitation of this phenomenon by viruses to gain access to the cell interior is a satisfying explanation for the IgSF CAM binding phenotype. However, the exploitation of these receptors by enveloped viruses that are thought to enter by fusion at the plasma membrane argues against this being the sole reason. Studies of reovirus infection in animal models show that JAM-A is required for viral dissemination through the bloodstream. It is postulated that this is related to the expression of JAM-A on endothelial cells, which may allow for transcytosis of virus into blood, or perhaps infection of the endothelium may lead to apical release of virus into the bloodstream. An alternative hypothesis is that IgSF CAM expression allows for viral dissemination from sites of primary infection in epithelial cells by transmission to responding migratory cells of the immune system.

It is hoped that a detailed understanding of processes of virus attachment and entry will provide researchers with new targets for anti-viral development. In particular, mechanisms found to be widespread among diverse groups of viruses, such as entry via IgSF CAM binding, are appealing targets as interventions have the potential for broad-spectrum activity. As targeting host systems, such as regulation of cell adhesion and immune cell transmigration, would seem to be a strategy liable to significant toxicity, detailed dissection of the manner in which viruses exploit these processes is critical.
